# Facile Organometallic Synthesis of Fe-Based Nanomaterials by Hot Injection Reaction

**DOI:** 10.3390/nano11051141

**Published:** 2021-04-28

**Authors:** Georgia Basina, Hafsa Khurshid, Nikolaos Tzitzios, George Hadjipanayis, Vasileios Tzitzios

**Affiliations:** 1 Institute of Nanoscience and Nanotechnology, NCSR Demokritos, 15310 Athens, Greece; georgia.basina@ku.ac.ae (G.B.); tz_nikos@yahoo.gr (N.T.); 2 Department of Chemical Engineering, Khalifa University, Abu Dhabi P.O. Box 127788, United Arab Emirates; 3 Department of Applied Physics and Astronomy, University of Sharjah, Sharjah 27272, United Arab Emirates; hkhurshid@sharjah.ac.ae; 4 Department of Physics and Astronomy, University of Delaware, Newark, DE 19711, USA; hadji@udel.edu

**Keywords:** iron, colloids, magnetic particles, core/shell, chemical synthesis

## Abstract

Fe-based colloids with a core/shell structure consisting of metallic iron and iron oxide were synthesized by a facile hot injection reaction of iron pentacarbonyl in a multi-surfactant mixture. The size of the colloidal particles was affected by the reaction temperature and the results demonstrated that their stability against complete oxidation related to their size. The crystal structure and the morphology were identified by powder X-ray diffraction and transmission electron microscopy, while the magnetic properties were studied at room temperature with a vibrating sample magnetometer. The injection temperature plays a very crucial role and higher temperatures enhance the stability and the resistance against oxidation. For the case of injection at 315 °C, the nanoparticles had around a 10 nm mean diameter and revealed 132 emu/g. Remarkably, a stable dispersion was created due to the colloids’ surface functionalization in a nonpolar solvent.

## 1. Introduction

Colloidal magnetic nanoparticles, with well-defined morphology and dimensionality, are of primary importance for both fundamental studies and prospective applications in many technological areas including magnetic storage devices [1,2], ferrofluids [3,4,5], magnetic resonance imaging [6,7,8,9,10], drug delivery [11,12,13,14], bio-separation [15,16,17], hyperthermia [18,19,20,21,22], sensing [23,24,25], and catalysis [26,27,28,29]. Amongst them iron-based magnetic materials including iron oxides [30,31,32], metallic iron [33,34,35], and iron alloys [36,37,38] have been extensively studied for many decades. A plethora of methodologies have been developed for their synthesis in both aqueous and non-aqueous media including co-precipitation [12,39], the polyol reaction [40,41,42], and thermolytic reactions at high temperature in an organic environment [43,44], which lead to the formation of monodispersed nanoparticles with controllable size, shape, and morphology [44,45,46,47]. Taking into consideration the synthesis of metallic iron nanoparticles and iron nanoalloys, well-defined morphologies in low dimension regimes and their stabilization against oxidation are challenges still to be overcome.

According to the literature, a common synthesis process of metallic Fe particles is the thermal decomposition of Fe(CO)_5_ in many different organic solvents such as octadecene [48,49] with a saturation magnetization up to 102 emu/g, octyl ether [33], and kerosene [50,51], showing 150 emu/g, while the reaction was taking place, after purification of the reactants, at room temperature in an oxygen free environment inside a glovebox. In addition, a different approach regarding the synthesis of metallic Fe nanoparticles has been reported by decomposing the iron carbonyl over noble metal seeds [33]. A frequent synthesis method is also the decomposition of iron(II) bis(trimethylsilyl)amide (Fe[NSi(CH_3_)_2_]_2_) [52] and the reduction of iron(III) acetylacetonate [53,54].

In this research study, we report on a facile organometallic approach based on the instantaneous thermal decomposition of Fe carbonyls in a multi-surfactant-based media, which leads to the synthesis of Fe-based nanomaterials from the Fe/Fe-oxide core/shell to hollow Fe-oxides.

## 2. Materials and Methods

### 2.1. Materials Synthesis

During a typical procedure, the oleyl amine, tri-octylphosphine, and oleic acid mixture is heated at 120 °C under gently magnetic stirring and degassed with 4% H_2_ in N_2_ for 1 h. Subsequently, the temperature rises to 180–315 °C, followed by the instantaneous injection of 1 mmol Fe(CO)_5_, which is dissolved in a similar molecular mixture composition under vigorous stirring. The reaction takes place for only 10 min. Afterward, the flask is removed carefully from the heating source and cooled down quickly by means of a water bath. The formed nanoparticles are precipitated by the addition of absolute ethanol and separated by the use of a Nd_2_Fe_14_B laboratory magnet. Several of these washes were conducted and the precipitated nanoparticles were dispersed and stored in hexane in order to prevent complete oxidation. It is noteworthy that all the chemicals were used without any purification or distillation step.

### 2.2. Analytical Methods

The crystal structure of the materials was determined using X-ray diffraction (XRD, Rigaku Ultima IV, University of Delaware, Newark DE) with CuKα radiation. The size and the morphology of the particles were determined using transmission electron microscopy (TEM, JEOL JEM-3010, University of Delaware, Newark DE) and the magnetic properties were measured with a 1 Tesla vibration sample magnetometer (VSM, Quantum Design, University of Delaware, Newark DE). Infrared spectra were taken with a Bruker, Equinox 55/S model, Fourier transform infrared (FT-IR) spectrometer (NCSR Demokritos, Athens, GR, USA), in the pellet KBr form.

## 3. Results and Discussion

**Figure 1:**
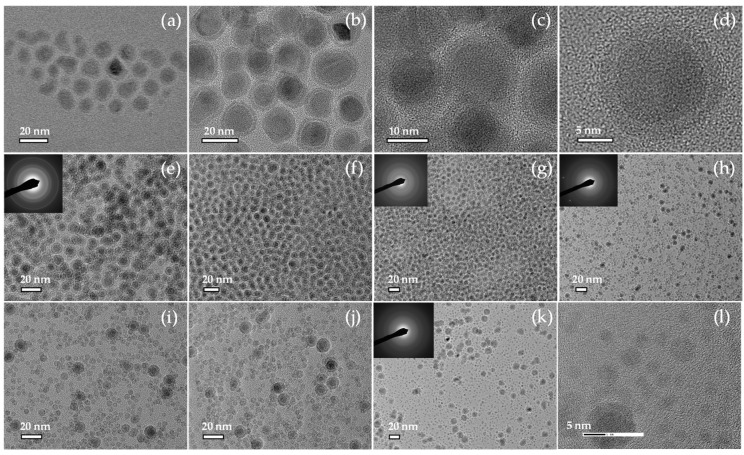
TEM images from Fe/Fe_3_O_4_ core-shell nanoparticles synthesized by hot injection at 315 °C (**a**), 285 °C (**b**–**d**), 260 °C (**e**), 240 °C (**f**), 220 °C (**g**), 200 °C (**h**–**j**), and 180 °C (**k**,**l**). Summarized data for the size distribution histograms were presented in Table S1.

**Figure 2:**
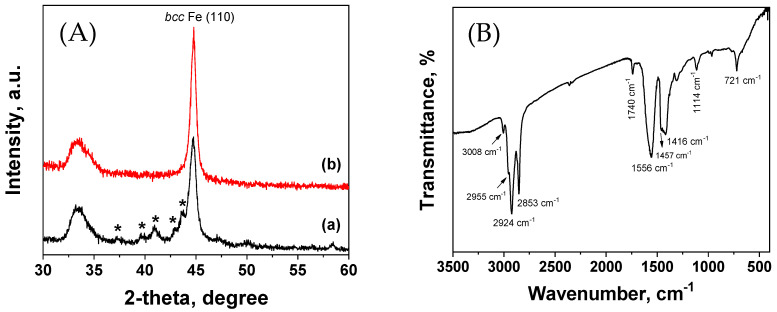
XRD patterns (**A**) of the core/shell Fe/Fe-oxide nanoparticles synthesized after carbonyl injection at 315 °C (**a**), and 285 °C (**b**), and the FTIR spectrum (**B**) of the particles were synthesized at 285 °C.

Figure 1 shows the transmission electron microscopy, TEM and High-resolution transmission electron microscopy, HR-TEM images together with the selective area diffraction, SAD, analysis (as inset) of the nanoparticles synthesized by Fe(CO)_5_ injection at the 180–315 °C temperature range. In the case of the carbonyl injection at 315 °C (Figure 1a), the nanoparticles had a core/shell morphology with irregular shapes and quite broad size distribution with 9.9 nm mean diameter (Figure S1), while decreasing the injection temperature at 285 °C (Figure 1b–d), they were spherical, uniform, and maintained the core/shell structure. Their overall size was around 12 nm, with a core of 9.5 ± 1.9 nm metallic Fe and 2.1 ± 0.3 nm shell (based on the size distribution histograms in Supplementary Materials Figure S2a,b). The core is single crystalline (ordered structure) while the shell is composed of randomly oriented grains of iron-oxide crystallite. The disordered structure in Figure 1d was an iron-oxide shell. The crystallite size has been estimated as about 14.2 nm by the Scherrer equation in Figure 2(Ab), taking into consideration the (110) peak, which corresponds to the *bcc* structure of metallic Fe. Figure 2A shows the XRD patterns received from the particles, which were synthesized after carbonyl injection at 315 °C (a), and 285 °C (b), respectively. In both cases, the XRD patterns in Figure 2A, showed a main diffraction peak at 44.7°, which belonged to the (110) plane of bcc Fe. However, when the particles were synthesized at a higher decomposition temperature, 315 °C, (Figure 2(Aa), the XRD pattern showed the presence of a secondary phase of iron carbide [55], which is indicated by an asterisk. The broad diffraction peak at 33.4° can be attributed to the substrate. The crystal structure of the rest materials was estimated by the selected area diffraction (SAD). The results are presented as insets in the corresponding TEM images in Figure 1. The synthesized nanoparticles between 220–260 °C demonstrate the *bcc* Fe structure very clearly. The prepared nanoparticles at 200 °C and 180 °C, whose rings are quite broad due to the formation of oxides as a dominant phase and in combination with their very small size, favor the amorphous phases. At lower injection temperatures and particularly in the range 260–220 °C, (Figure 1e–g), the particles were quite monodispersed with a core/shell morphology and with even smaller particles sizes. Specifically, the nanoparticles prepared at 260 °C had an overall size of ~8.3 nm with a metallic Fe core of 6.27 ± 1.01 nm and shell of 2.02 ± 0.3 nm (Figure S2c,d in the Supplementary Materials). Similarly, the particles synthesized at 240 °C have an overall particle size ~8.1 nm, with a metallic Fe core of 5.69 ± 0.98 nm and a shell of 2.37 ± 0.36 nm (Figure S2e, f in the Supplementary Materials), while at 220 °C, the nanoparticles possessed even smaller core sizes of 4.9 ± 0.8 nm, as shown in Figure S3 (Supplementary Materials). Finally, as the injection temperature took place in the 180–200 °C temperature range, the synthesized nanomaterials consist of two clearly distinguished kinds of particles. Very small particles of iron oxides are presented predominantly, while larger core/shell structure particles of probably Fe/Fe-oxide have also been demonstrated. The only difference between the synthesized materials at 200 °C and 180 °C was the nature of the iron oxide particles, which revealed a hollow and solid structure, respectively. The hollow ones were a result of the Kirkendall effect during the in situ oxidation of tiny metallic iron particles, while the formation of solid particles at 180 °C indicates that the oxidation followed a different route. Overall, the formation of iron oxide phases, either as a shell or as individual particles, was due to the presence of dissolved oxygen in the reaction mixture. Concisely, we should emphasize that when the carbonyl injection took place at reaction temperatures greater than 285 °C, the particles demonstrated a core/shell morphology with a metallic Fe core and iron oxide shell while iron carbides were presented. On the other hand, it was observed that the formation of monodispersed core/shell nanoparticles is favored at moderate injection temperatures. Carbonyl injection below 200 °C leads to the formation of a small amount of core/shell type particles with a metallic core and individual tiny iron oxides as hollow or solid particles.

The nanoparticle’s surface grafting, which is responsible for their dispersibility in non-polar solvents, was confirmed by infrared spectroscopy. Figure 2B shows the Fourier transform infrared, (FT-IR), spectra of Fe/Fe-oxide nanoparticles, which were synthesized at 285 °C. The spectrum showed strong absorptions at 2955, 2924, and 2853 cm^−1^ due to the CH_2_ stretching mode of the aliphatic chains, whereas a weak but characteristic band at 3008 cm^−1^ was due to the cis −HC=CH− arrangement in both the oleic acid and oleyl amine molecules. Carboxylic groups from the n-aliphatic acids coordinate to surface metal centers by monodentate and bidentate modes. The band at 1740 cm^−1^ was due to carbonyl symmetrical stretching vibration, indicating monodentate mode, while the existence of two bands at 1556 cm^−1^ (*asym*) and 1416 cm^−1^ (*sym*), indicated bidentate attachment of the oleate anion onto the surface. Bidentate binding was further reinforced from the difference Δν = ν_as_ − ν_s_ (1556−1416 = 140 cm^−1^). The absorption at 1457 cm^−1^ was due to the CH_2_ scissor mode. The broad band in the 3320 cm^−1^ region was attributed to the *ν*(NH) stretching mode. Finally, the adsorption band at 721 cm^−1^ can be ascribed from the methyl rock vibration and appeared only in long chain aliphatic molecules. The adsorption bands around 1114 cm^−1^ and 1170 cm^−1^ were probably due to the C–P stretching modes of TOP molecules. Subsequently, the nanoparticles’ surface was modified from all the capping agents that were present in the reaction mixture. However, in principle, the low intensity adsorption bands in the case of the phosphines should be taken into account.

**Figure 3:**
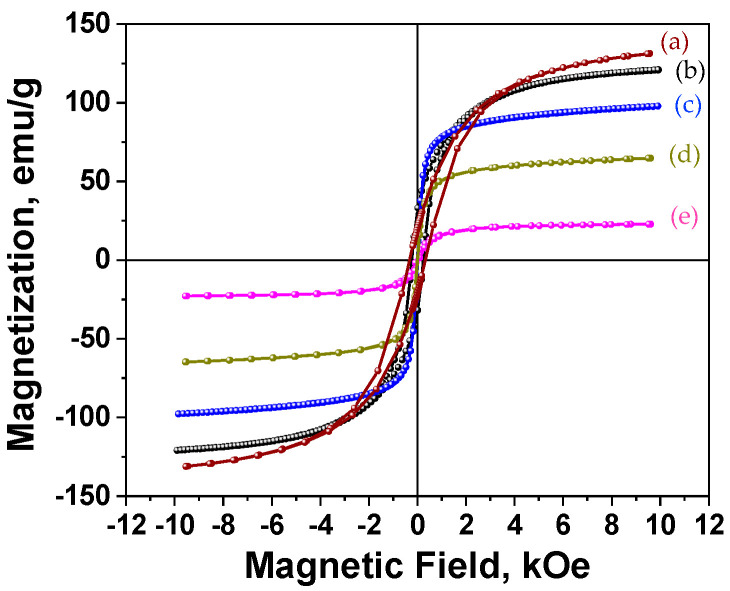
Room temperature hysteresis loops of the Fe-based core/shell nanoparticles synthesized at 315 °C (**a**), 285 °C (**b**) 260 °C (**c**) 240 °C (**d**) and 180 °C (**e**).

The magnetic properties of the corresponding nanomaterials were determined using a 1 T vibrating sample magnetometer (VSM). Figure 3 shows the room temperature magnetic hysteresis loops for the synthesized particles by hot injection at various temperatures. The prepared nanoparticles at 315 °C and 285 °C exhibited weak ferromagnetism with the 330 Oe and 230 Oe coercive field, respectively. The ferromagnetic behavior was probably due to magnetic interactions between the metallic iron cores and the well crystalline shells (due to the high reaction temperature), together with the presence of other phases (e.g., carbides). In contrast, the nanoparticles synthesized at lower temperatures demonstrated superparamagnetic behavior. The magnetization values at 1 Tesla ranged from 132 emu/g (injection at 315 °C) to 22.9 emu/g (reaction at 180 °C). The magnetization value at 1 T progressively decreased from 97.7 to 64.8 and 22.9 emu/g, for reaction temperatures of 260 °C, 240 °C, and 180 °C, respectively. The significant drop in the magnetization at the lowest reaction temperature was attributed to the formation of a large number of oxides along with the size effects.

**Figure 4:**
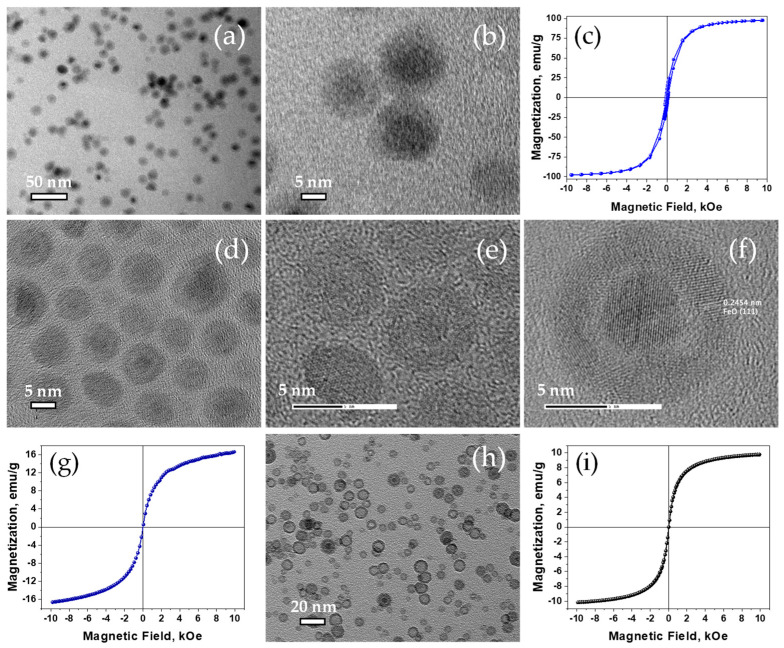
TEM images of the ~12 nm water soluble TMAOH functionalized Fe/Fe-oxide core/shell nanoparticles (**a**,**b**), and their room temperature magnetic hysteresis loop (**c**). TEM images of ~7 nm Fe/Fe-oxide synthesized with injection at 240 °C next day (**d**–**f**) and the corresponding room temperature magnetic hysteresis loop (**g**). TEM image of the hollow nanoparticles arising from the complete oxidation of the corresponded core/shell ones (**h**) and their room temperature magnetic hysteresis loop (**i**).

The resistance against further oxidation of the core/shell nanoparticles was studied through their transformation to water soluble nanoparticles by using tetra methyl ammonium hydroxide, TMAOH, as a transfer agent, according to a similar literature protocol procedure [30,56]. The particles that were synthesized by injection at temperatures higher than 285 °C retained their core/shell structure, (TEM images in Figure 4a,b), and their high magnetization at 1 Tesla, 99 emu/g, (Figure 4c), after one week of the phase transfer. In contrast, the smaller particles transformed very quickly to yolk/shell (Figure 4d–f), with a significant drop in their magnetization value, (Figure 4g) and finally altered to hollow Fe-oxides (Figure 4h) with a magnetization value at the level of 10 emu/g (Figure 4i).

## 4. Conclusions

In summary, metallic Fe-based nanoparticles were synthesized by the facile hot injection approach of Fe(CO)_5_ in a surfactant mixture that was used as received, without conducting a purification process. The injection temperature is very crucial for the nature of the formed nanoparticles. Following an injection procedure at temperatures higher than 220 °C, the synthesized nanoparticles possessed an Fe/Fe-oxide core/shell morphology; while at lower temperatures, hollow morphology or solid ultra-small Fe-oxides nanoparticles, were received as the dominant product. Furthermore, the Fe/Fe-oxides particles with an overall mean particle size of ~10 nm, revealed a 132 emu/g magnetization value at a 1 T applied field. These nanoparticles were quite stable against complete oxidation in contrast to the smaller ones, which were easily converted to yolk/shell and finally to hollow iron oxides. The stability against oxidation of the larger particles is attributed to the size effects, along with the presence of other phases such as carbides.

## Supplementary Materials

The following are available online at https://www.mdpi.com/article/10.3390/nano11051141/s1, Figures S1–S3: Size distribution histograms of the Fe/Fe-oxides synthesized at various injection temperatures. Table S1: Summarized data received from Histograms in Figures S1–S3.
