# Supplementary material for: Facile Organometallic Synthesis of Fe-Based Nanomaterials by Hot Injection Reaction
Source: Nanomaterials (Basel). 2021 Apr 28;11(5):1141. doi: 10.3390/nano11051141 (PMC8145410; doi:10.3390/nano11051141)
Supplement: Supplementary file 1 [file nanomaterials-11-01141-s001.zip › nanomaterials-1192082-supplementary.pdf]

Supporting Information

Facile Organometallic Synthesis of Fe-Based Nanomaterials by Hot Injection Reaction

Georgia Basina [1,2], Hafsa Khurshid [3,4], Nikolaos Tzitzios [1], George Hadjipanayis [4] and Vasileios Tzitzios [1,*]

1 Institute of Nanoscience and Nanotechnology, NCSR Demokritos, 15310 Athens, Greece; georgia.basina@ku.ac.ae (G.B.); tz_nikos@yahoo.gr (N.T.)
2 Department of Chemical Engineering, Khalifa University, Abu Dhabi P.O. Box 127788, United Arab Emirates
3 Department of Applied Physics and Astronomy, University of Sharjah, Sharjah 27272, United Arab Emirates; hkhurshid@sharjah.ac.ae
4 Department of Physics and Astronomy, University of Delaware, Newark, DE 19711, USA; hadji@udel.edu
* Correspondence: v.tzitzios@inn.demokritos.gr; Tel.: +30-210-650-3319

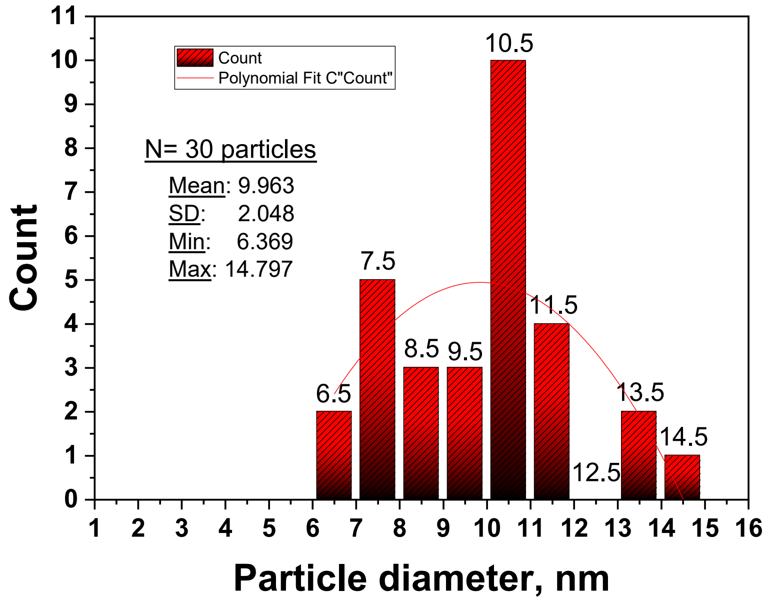

Figure S1. Histogram of overall particle (Fe/Fe-oxides) size with core/shell morphology derived from the TEM image in Figure 1a, which corresponds to nanoparticles synthesized by hot injection of $Fe(CO)_5$ at 315 °C.

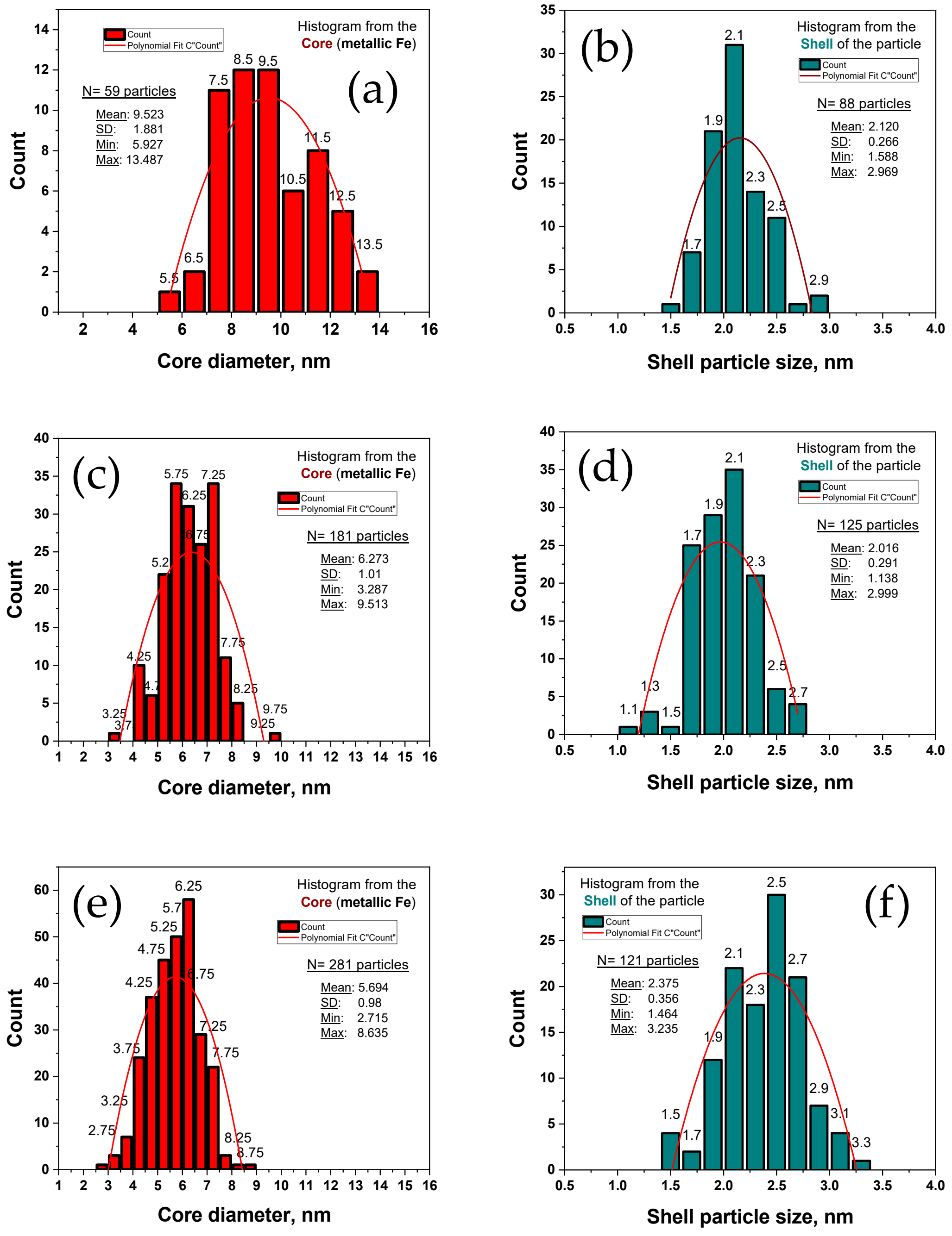

Figure S2. Histogram of core size (on the left) and shell (on the right) from Fe/Fe-oxides nanoparticles with a core/shell morphology, derived from: a,b: the TEM images of Figure 1(b,c,d) for the nanoparticles synthesized by hot injection of $Fe(CO)_5$ at 285 °C (a, b), from c, d: the TEM image of Figure 1(e) for the nanoparticles synthesized at 260 °C (c, d), and from e, f: the TEM image of Figure 1(f), d for the nanoparticles synthesized by hot injection of $Fe(CO)_5$ at 240 °C (e, f).

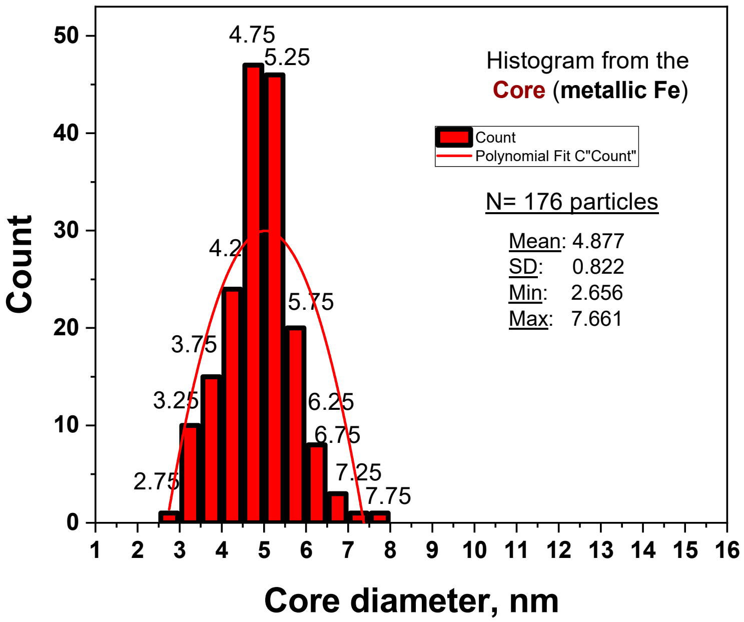

Figure S3. Histogram of metallic Fe/core particles in the Fe/Fe-oxides size with core/shell morphology derived from the TEM image in Figure 1g, which corresponds to nanoparticles synthesized by hot injection of $Fe(CO)_5$ at 220 °C.

Table S1. Summarized data extract from Histograms in Figure S1-S3.

**Sample**	**Reaction Temperature**	**Particles**	**Parameters obtained by the histograms**[1]			**Mean size (nm)**		
**Figure S1-3.**	**T** **hot injection (°C)**	**N**	**Minimum Size (nm)**	**Maximum Size (nm)**	**SD**	**Overall particle (Fe/Fe-oxides)**	**Core (metallic Fe)**	**Shell**
S1	315	30	6.369	14.797	2.048	**9.963**	-	-
S2 (a,b)	285	59	5.927	13.478	1.881	-	**9.523**	2.12±0.266
S2 (c,d)	260	181	3.287	9.513	1.01	-	**6.273**	2.016±0.291
S2 (e,f)	240	281	2.715	8.635	0.98	-	**5.694**	2.375±0.356
S3	220	176	2.656	7.661	0.822	-	**4.877**	-

[1] Correspond to overall particle size (Fe/Fe-oxides) or to the core (metallic Fe) of the particles.
